# Negative Symptoms of Psychosis Correlate with Gene Expression of the Wnt/**β**-Catenin Signaling Pathway in Peripheral Blood

**DOI:** 10.1155/2013/852930

**Published:** 2013-01-01

**Authors:** Chad A. Bousman, Stephen J. Glatt, Sharon D. Chandler, James Lohr, William S. Kremen, Ming T. Tsuang, Ian P. Everall

**Affiliations:** ^1^Department of Psychiatry, University of Melbourne, Royal Melbourne Hospital, Level 1 North, Parkville, VIC 3050, Australia; ^2^Department of Psychiatry and Behavioral Sciences, SUNY Upstate Medical University, Syracuse, NY 13210, USA; ^3^Department of Psychiatry, University of California, San Diego, CA 92093, USA; ^4^Department of Psychiatry, VA San Diego Healthcare System, La Jolla, CA 92161, USA; ^5^Department of Epidemiology and Psychiatry, Harvard University, Cambridge, MA 02138, USA

## Abstract

Genes in the Wnt (wingless)/**β**-catenin signaling pathway have been implicated in schizophrenia pathogenesis. No study has examined this pathway in the broader context of psychosis symptom severity. We investigated the association between symptom severity scores and expression of 25 Wnt pathway genes in blood from 19 psychotic patients. Significant correlations between negative symptom scores and deshivelled 2 (DVL2) (*r*_adj_ = −0.70; *P* = 0.0008) and glycogen synthase kinase 3 beta (GSK3B) (*r*_adj_ = 0.48; *P* = 0.039) were observed. No gene expression levels were associated with positive symptoms. Our findings suggest that the Wnt signaling pathway may harbor biomarkers for severity of negative but not positive symptoms.

## 1. Introduction

The Wnt (wingless)/*β*-catenin signaling pathway is involved in a multitude of neuronal processes including regulation of synaptogenesis, synapse specificity, axon guidance, dendrite development, and overall brain development [1]. Numerous genes involved in the Wnt/*β*-catenin signaling pathway have been implicated in the pathogenesis of several neurodevelopmental disorders including schizophrenia, bipolar disorder, and autism spectrum disorders [2]. While psychosis is a key feature of schizophrenia and present in many cases of bipolar disorder, to date the relationship between the Wnt/*β*-catenin signaling pathway and symptom severity in psychosis has not been investigated. Thus, we sought to correlate blood-based gene expression of the Wnt/*β*-catenin signaling pathway with negative and positive symptom severity indices of patients with a history of psychosis (i.e., schizophrenia or bipolar disorder). We were guided by previous research that has examined the relationship between psychosis symptom severity and other gene expression pathways in peripheral blood [3, 4] and has demonstrated the utility of blood as a source of biomarkers for brain disorders [5].

## 2. Materials and Methods

Clinical and gene expression data from 19 subjects (Table 1) meeting DSM-IV criteria for at least one episode of psychosis were acquired from a larger gene expression study of blood-based biomarkers of schizophrenia and bipolar disorder (GEO Accession number: GSE18312). All subjects were currently taking psychotropic medication and were excluded if any of the following were present: (1) substance abuse or dependence in the past year, (2) neurologic problems (e.g., stroke, meningitis), (3) systemic medical illnesses (e.g., heart disease, diabetes), (4) history of head injury with documented loss of consciousness lasting longer than 10 minutes, (5) pregnancy, or (6) physical disabilities. More details on the recruitment, inclusion, and exclusion criteria as well as procedures used to diagnose psychosis were described previously [6]. Whole blood samples (10 mL) were collected into EDTA-coated collection tubes the morning after subjects fasted overnight. Preparation of blood samples, separation, and lysis of PBMCs, extraction, purification, and hybridization of RNA, quantification of expression levels on GeneChip Human Exon 1.0 ST Arrays (Affymetrix, Inc.; Santa Clara, CA), and quality-control procedures were all performed by standard methods, which are described in greater detail elsewhere [6, 7]. All study procedures were approved by the Institutional Review Board at University of California, San Diego.

### 2.1. Negative and Positive Symptom Severity Measurement

The Scales for the Assessment of Positive and Negative Symptoms (SAPS-SANS) [8] were administered to all subjects by a trained Master's-level research assistant and later reviewed by two independent doctoral-level clinicians to ensure accurate scoring. The SAPS assesses four distinct positive symptom subcategories (i.e., hallucinations, delusions, bizarre behavior, and thought disorder), whereas the SANS assesses five negative symptom subcategories (i.e., affective flattening, alogia, avolition, anhedonia-asociality, and attention) on a severity scale of zero (none) to five (severe). For the current study, the four and five subcategory scores were averaged to create global scores for positive and negative symptoms, respectively (Table 1). 

### 2.2. Gene Selection

The number of genes that populate the Wnt/beta-catenin signaling pathway is continually changing, and a consensus on the number has not been achieved. We focused on 25 of the most well-characterized genes involved in the Wnt signaling pathway, based on the Kyoto Encyclopedia of Genes and Genomes database (http://www.genome.jp/kegg/). These genes included v-akt murine thymoma viral oncogene homolog (*AKT1*), adenomatous polyposis coli (*APC*), axin 1 (*AXIN1*), catenin beta 1 (*CTNNB1*), disrupted in schizophrenia 1 (*DISC1*), Dickkopf homologs 1, 3, and 4 (DKK1, 3, 4), disheveled homologs 1–3 (*DSL1–3*), frizzled homologs 1–8 (*FZD1–8*), glycogen synthase kinase 3 alpha (*GSK3A*) and beta (*GSK3B*), low density lipoprotein receptor-related proteins 5 (*LRP5*) and 6 (*LRP6*), transcription factor 4 (TCF4), and wingless-type MMTV integration site family member 1 (*WNT1*). Although many genes that were not included may provide further insight into the Wnt signaling pathway as it relates to psychotic symptoms, we were careful to select both up- and downstream genes within the pathway in an effort to provide an adequate proxy of Wnt pathway functioning. 

### 2.3. Statistical Analysis

Data preparation and analysis procedures have been described in detail elsewhere [3]. Briefly, Spearman's correlations were conducted between expression intensities for each of the 25 selected Wnt pathway genes and SANS-SAPS global severity scores, adjusting for gender, ethnicity, age, education, current smoking (yes/no), and past six-month substance use (yes/no). Due to the inflated probability of committing type-I errors in this study a Bonferroni-adjusted alpha threshold of 0.05/50 = 0.001 was used. 

## 3. Results

**Figure 1:**
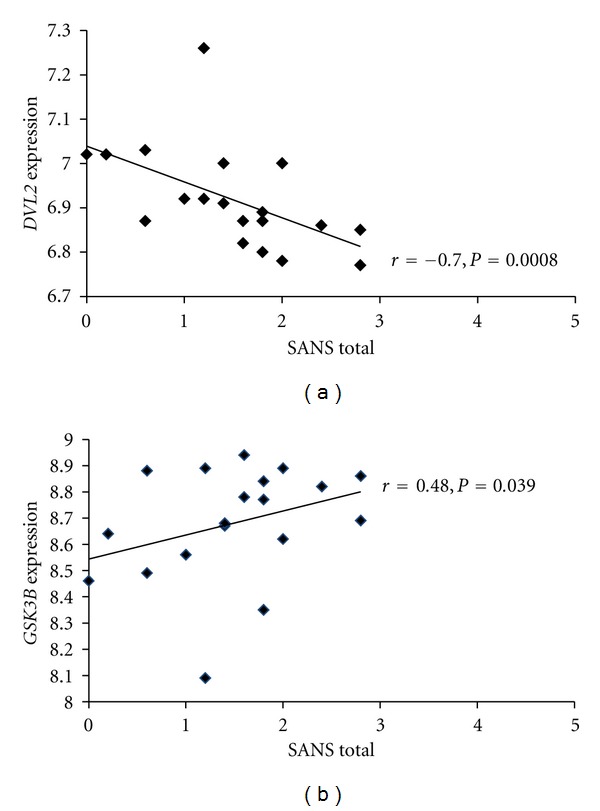
Correlations between severity of negative symptoms (SANS) and expression of two Wnt Pathway genes, dishevelled 1 (*DVL1*) and glycogen synthase kinase 3 beta (*GSK3B*). Higher scores on the SANS reflect greater severity.

Two (*DVL2 and GSK3B*) of the 25 WNT signaling pathway genes examined were nominally correlated with scores on the SANS however, only *DVL2* remained significant after Bonferroni correction. *DVL2* showed a negative correlation whereas, *GSK3B* was positively correlated with the SANS (Figure 1). Additionally, *TCF4* showed a trend toward negative correlation (*r* = −0.43, *P* = 0.060) with negative symptoms. *Post hoc* exploration of the four subscales of the SANS revealed significant negative correlations between *DVL2* expression and affective flattening (*r* = −0.55, *P* = 0.015) and alogia (*r* = −0.65, *P* = 0.003) severity. *GSK3B* expression was positively correlated with alogia (*r* = 0.60, *P* = 0.007) only. None of the 25 gene transcripts examined significantly correlated with severity scores on the SAPS. We were unable to perform qRTPCR validation for our genes of interest due to inadequate mRNA quantities although previous work with the current sample has successfully validated genes using qRTPCR [6]. 

## 4. Discussion

Our results show that lower expression of *DVL2* and to a lesser extent higher expression of *GSK3B* are associated with more severe negative but not positive symptoms in psychosis. To our knowledge, no study has examined the association between psychosis symptom severity and gene expression in the Wnt signaling pathway. Furthermore, we are unaware of any study linking *DVL2* gene expression to psychosis or other major psychiatric disorders, although numerous studies have implicated *GSK3B* in the pathogenesis of schizophrenia as well as antipsychotic drug action [1]. 

As the current study was designed to identify potential biomarkers in peripheral blood for psychosis symptom severity, we do not assume that our findings represent the underlying biological mechanisms by which clinically observed symptom severity emerges. However, our results are aligned with those of Emamian and associates [9] who reported decreased phosphorylation of *GSK3B* at Ser9 in peripheral lymphocytes and frontal cortex of individuals with schizophrenia, suggesting increased *GSK3B* activity. Our results are further supported by numerous *in vitro *studies demonstrating that disheveled homologues, such as *DVL2,* inhibit *GSK3B* phosphorylation of beta-catenin [10, 11] as well as work carried out within our laboratory demonstrating reduced levels of beta-catenin in the hippocampus of schizophrenic subjects [12], indicative of increased activity of *GSK3B*. However, contrary findings have been reported by us and others showing no difference in *GSK3B* mRNA levels in lymphocytes [13] and protein levels in the prefrontal cortex [14] compared to controls. 

Several lines of investigation have reported links between genes associated with the Wnt signaling pathway and schizophrenia [2, 15], most of which were included in the current analysis. Only two of the 25 genes examined were nominally associated with psychosis symptom severity. Our ability to uncover significant gene correlates involved in the Wnt signaling pathway may have been inhibited by several limitations within our study. First, the sample was small and may have prohibited us from detecting correlations that would have attained statistical significance in a larger sample. In fact, *TCF4, *one of only three genes correlated with schizophrenia at the whole genome level [16], had a large expression effect (*r* = −0.43; data not shown) [17] for negative symptoms suggesting that a significant correlation may have been observed in a larger sample. Second, we included subjects with schizophrenia or bipolar disorder with a history of psychosis. This decision was made on the assumption that psychosis symptoms in both disorders are similar and that an increase in the sample size and subsequent power would outweigh any potential statistical noise introduced by combining the two disorders. Third, all of the 19 participating subjects were taking psychotropic medication, which could have artificially reduced or inflated our observed correlations with symptom severity. Data from the Stanley Medical Research Institute Online Genomics Database (http://www.stanleygenomics.org/) suggest that *DVL2* is upregulated in postmortem brains of patients with greater than 5000 mg of lifetime antipsychotic exposure. In addition, lithium [18] as well as clozapine, olanzapine, risperidone, quetapine, and ziprasidone are known to inhibit *GSK3B* in the mouse and rat brain [19, 20]. More than half (53%, *n* = 10) of the 19 subjects in the current study were taking one or more of these medications at the time of assessment but post hoc adjustment for presence of one of these medications showed no difference in our reported results. Therefore, these results serve as preliminary evidence that the Wnt signaling pathway may harbor biomarkers for (primarily negative) symptom severity in psychotic disorders. Future longitudinal studies designed *a priori* to examine gene expression prior to, during, and after a psychotic episode and initiation of pharmacological treatment would clarify the biomarker potential of this pathway for psychosis symptom severity. Finally, it remains unclear whether expression in peripheral blood accurately reflects that found in the brain [21]. However, it is not a requirement for a biomarker to be linked etiologically to the phenotype of interests. Future postmortem expression studies with access to antemortem clinical data could further strengthen our results. In addition, future research examining expression of a larger pool of Wnt signaling genes including *DVL2* and *GSK3B* using more sensitive techniques (e.g., qRTPCR) is also warranted. If replicated, these results provide potential first steps toward development of objective tools for characterizing psychosis symptom profiles in clinical practice.

## 5. Conclusion

Our results support previous work and provide preliminary data suggesting the Wnt signaling pathway as one of potentially many pathways harboring transcriptomic biomarkers for negative symptom severity in psychosis. 

## Figures and Tables

**Table 1:** Sample characteristics.

Variable	Total (*n* = 19)
Age: mean years (sd)	43 (9)
Sex: female *n* (%)	6 (32)
Education: mean years (sd)	13 (2)
Ancestry: *n* (%)	
European	10 (53)
African	6 (32)
Hispanic	2 (10)
Asian	1 (5)
Current smoker: *n* (% yes)	13 (68)
Past 6-month substance use: *n* (% yes)	3 (16)
Current medication use: *n* (% yes)	
Antipsychotic	16 (84)
Mood stabilizer	7 (37)
History of psychosis: *n* (% yes)	19 (100)
Positive symptoms: mean score (sd)^1^	
Hallucinations	2.8 (1.7)
Delusions	2.7 (1.3)
Bizarre behaviors	0.3 (0.5)
Formal thought disorder	0.5 (0.8)
Positive symptom total (SAPS)	1.6 (0.6)
Negative symptoms: mean score (sd)^1^	
Affective flattening	1.9 (1.4)
Alogia	1.2 (1.5)
Apathy	2.2 (1.5)
Anhedonia	2.0 (1.7)
Attention	0.3 (0.6)
Negative symptom total (SANS)	1.5 (0.8)

^1^Higher scores reflect greater severity (range 0–5).

SAPS: Scale for the Assessment of Positive Symptoms.

SANS: Scale for the Assessment of Negative Symptoms.
